# Identification of Prognostic Factors in Patients With Streptococcus Bloodstream Infection

**DOI:** 10.3389/fmed.2022.832007

**Published:** 2022-04-26

**Authors:** Xiaoguang Duan, Ruifang Zhang, Xiaojuan Zhang, Xianfei Ding, Tongwen Sun

**Affiliations:** General ICU, Henan Key Laboratory of Critical Care Medicine, Zhengzhou Key Laboratory of Sepsis, Henan Engineering Research Center for Critical Care Medicine, The First Affiliated Hospital of Zhengzhou University, Zhengzhou, China

**Keywords:** streptococcus, bloodstream infection, prognostic, antibiotics, 60-day mortality

## Abstract

**Aim:**

The purpose of this study was to explore prognostic factors of bloodstream infections (BSIs), a common severe infection and a major cause of mortality worldwide, so as to construct a prognosis model of patients with BSI.

**Materials and Methods:**

Clinical and biochemical test data were obtained retrospectively from the medical records of 562 patients with BSI who had been treated at a single center; the end point was 60 days of all-cause death. The chi-square test was used to compare the mortality of patients grouped by the types of antibiotic treatment. The logistic regression analysis was adopted to identify prognostic factors; the Kaplan–Meier survival curve and log-rank test were conducted to compare the survival rate of patients with different prognostic factors; the receiver operating characteristic (ROC) curve was used to estimate the predictive value of different prognostic factors.

**Results:**

Of the 562 patients, 455 survived (80.96%), and 107 died (19.04%). The mortality rate of patients treated with a combination of antibiotics (25.40%) was higher than that treated with a single antibiotic (15.82%). Univariate analysis identified 19 prognostic factors for patients with BSI, including gender, age, diabetes, malignant tumor (non-blood system), total hospitalization time, alanine aminotransferase, aspartate aminotransferase, total protein, albumin, total bilirubin, direct bilirubin, creatinine, ratio of granulocytes, fibrinogen, D-dimer, platelet, C-reactive protein, shock, and respiratory failure (*P* < 0.05). Multivariate analysis indicated that albumin (odds ratio [OR] = 0.94, 95% confidence interval [CI]: 0.89–0.99), fibrinogen (OR = 0.61, 95%CI: 0.46–0.82), shock (OR = 16.61, 95%CI: 7.00–39.41), and respiratory failure (OR = 47.53, 95%CI: 19.93–133.64) were independent factors. The combination of four indicators demonstrated a favorable predictive value for the 60-day outcome of patients with BSI, with an area under the ROC of 0.96 (95%CI: 0.94–0.99), sensitivity of 90.65%, specificity of 94.95%, and accuracy of 94.13%.

**Conclusions:**

Shock, respiratory failure, albumin, and fibrinogen are potential independent prognostic factors for 60-day mortality.

## Introduction

Bloodstream infection (BSI) is a common severe infection and a major cause of mortality worldwide; its morbidity is comparable to that of acute myocardial infarction and trauma (1, 2). BSIs can be caused by bacteria, fungi, viruses, and protozoa, but among the four groups of pathogens, bacteria account for most BSIs. Streptococci rank in the top 10 bacterial pathogens causing BSI. Streptococcus comprises common bacteria of pyogenic cocci and includes three different species: α-hemolytic streptococcus, β-hemolytic streptococcus, and γ-streptococcus (non-hemolytic). β-hemolytic streptococcus is an important invasive infection in humans, causing skin and soft tissue infection, infective endocarditis, arthritis, bacteremia, and so forth (3, 4). Streptococcus BSI has a poor prognosis. The most dangerous outcome is toxic shock syndrome complicated by multiple organ dysfunctions, which has a mortality rate of 50–70% (5, 6). Therefore, it is necessary to explore more reliable indicators for detecting and evaluating the inflammatory response, infection, and sepsis so that the appropriate treatment measures can be implemented, ultimately improving survival rates and prolonging the survival time of patients with streptococcus BSI.

An epidemiological study including 9,268 cases of BSI in southeast Sweden indicated that age, gender, and cancer were significantly associated with 30-day mortality (7). Based on an analysis of 147 patients with BSI, Samuel et al. reported that factors associated with the increased risk of BSI developing severe streptococcal infection risk included older age, neutropenia at the onset BSI, and source of respiratory infection (8). Alessander et al. revealed 13 potential proteins correlated with mortality and persistent bacteremia caused by *Staphylococcus aureus* and identified interleukin (IL)-8 and CCL2 as the strongest individual predictors of mortality (9). However, there have been few studies on prognostic factors (mainly including biochemical indicators and clinical characteristics) of patients with streptococcus-associated BSI.

**Figure 1:**
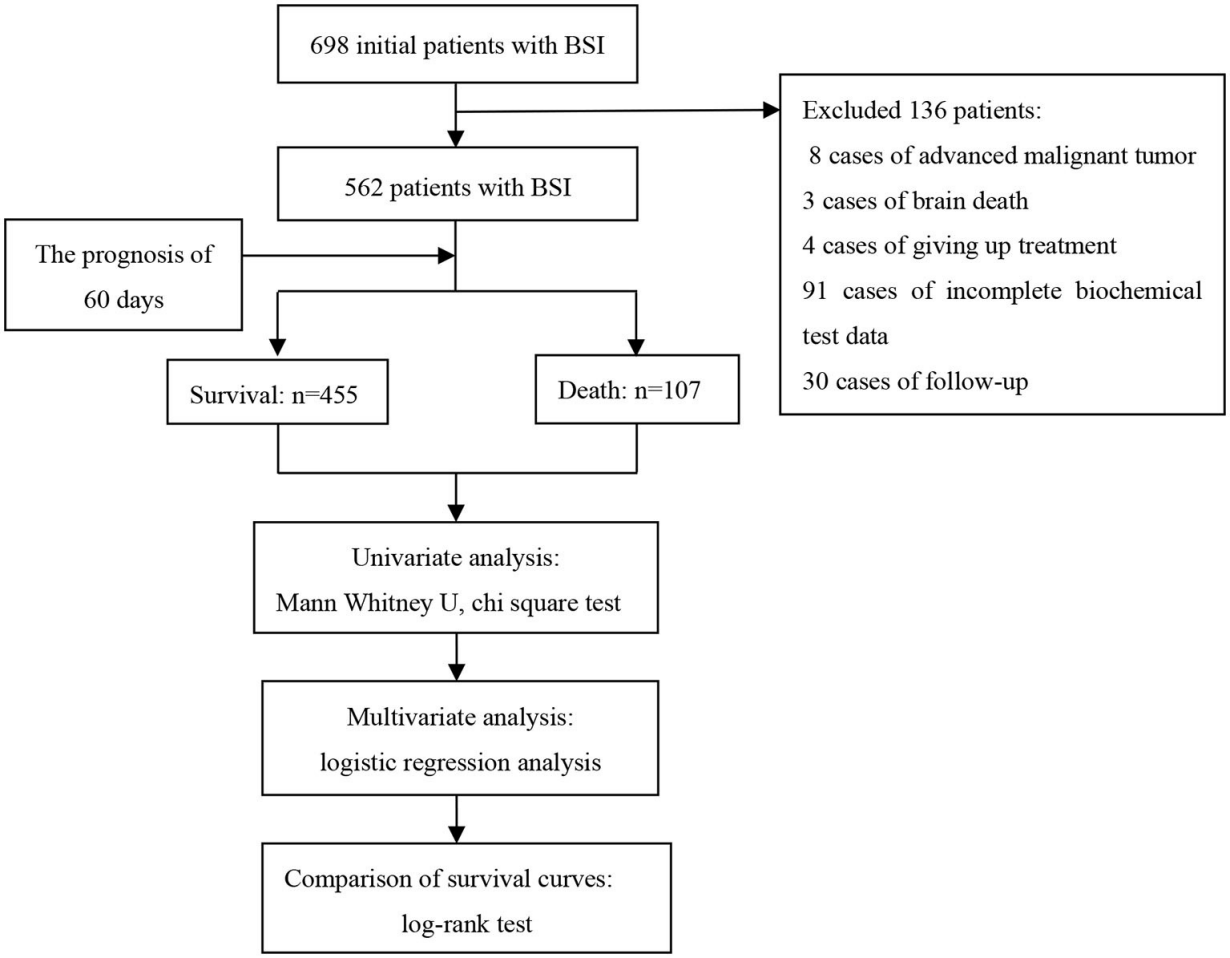
Study design.

In this study, we explored the prognostic factors of patients with streptococcus BSI based on a prospective follow-up cohort and constructed a prognosis model of biochemical indicators and clinical characteristics. The study design is illustrated in Figure 1.

## Materials and Methods

### Patients in the Study

In this study, 698 patients with BSI treated in the First Affiliated Hospital of Zhengzhou University from January 2015 to December 2019 were selected as research objects. Finally, according to the inclusion and exclusion criteria, 562 patients with BSI were included in this study. The inclusion criteria were that blood culture and clinical symptoms (e.g., a clear source of infection, elevated body temperature, elevated infection indicators, and simultaneous culture of streptococcus in other parts) supported streptococcus BSI. Patients with a terminal-stage malignant tumor, brain death, loss of follow-up, abandonment of treatment, and/or data loss were excluded.

### Information Collection

Patients with BSI were followed up by telephone from January 2020 to March 2020, and the end point was 60 days of all-cause death. According to a prognosis of 60 days, the patients were divided into death group and survival group. The clinical and biochemical test data of all subjects were collected from a comprehensive electronic medical record using standardized data collection tables, which mainly included gender, age, past history, respiratory failure, shock, hospitalization time, alkaline phosphatase, alanine aminotransferase, indirect bilirubin, fibrinogen, D-dimer, platelet, C-reactive protein, and other indicators within 48 h after positive blood culture. Approval for this study was obtained from the Medical Ethics Committee of the First Affiliated Hospital of Zhengzhou University (Ethical number: 2020-KY-157).

### Statistical Analysis

IBM SPSS statistics software (version 21.0) and GraphPad Prism 6.0 were used for data analysis. Categorical data were expressed as percentages. Continuous data were described using the mean and standard deviation or median and quartile. The Kolmogorov–Smirnova test was used to determine whether the data had a normal distribution. The *t-*test was employed to compare the data with a normal distribution, and the Mann–Whitney *U* test was applied for data that did not conform to the normal distribution. The classification data were compared using chi-square or Fisher's exact test. The logistic regression analysis was adopted to identify prognostic factors. The Kaplan–Meier survival curve and log-rank test were performed to compare the survival rate of patients with different prognostic factors. The receiver operating characteristic (ROC) curve was used to estimate the predictive value of different prognostic factors. All *P* values were determined based on the two-tailed test, and *P* < 0.05 was considered significant.

## Results

### Characteristics of the Study Population

Among 698 patients with BSI, 136 cases were excluded, including 8 cases of end-stage malignant tumor, 3 cases of brain death, 4 cases of giving up treatment, 91 cases of incomplete biochemical test data, and 30 cases of patients due to loss of follow-up. The follow-up rate was 94.9% (562/592). Thus, 562 eligible patients were used for statistical analysis, including 337 males and 225 females. The patients were divided into survival group (*n* = 455 [80.96%]) and death group (*n* = 107 [19.04%]) based on the outcome of 60 days. The ages of the survival group and death group were 44.4 ± 22.1 and 51.4 ± 25.0 years, respectively. The basic characteristics of the 562 participants are summarized in Table 1.

**Table 1:** Basic characteristics of 562 patients with streptococcal bloodstream infection.

**Variable**	**Survival group** **(*n* = 455)**	**Death group** **(*n* = 107)**
**Sex (** ***n*** **, %)**		
Male	263 (57.8)	74 (69.2)
Female	192 (42.2)	33 (30.8)
Year (x ± SD)	44.4 ± 22.1	51.4 ± 25.0
**Hypertension (** ***n*** **, %)**		
no	407 (89.5)	90 (84.1)
yes	48 (10.5)	17 (15.9)
**Diabetes (** ***n*** **, %)**		
no	409 (89.9)	84 (78.5)
yes	46 (10.1)	23 (21.5)
**Coronary heart disease (** ***n*** **, %)**		
no	439 (96.5)	103 (96.3)
yes	16 (3.5)	4 (3.7)
**Malignant tumor (non-blood system) (** ***n*** **, %)**		
no	406 (89.2)	84 (78.5)
yes	49 (10.8)	23 (21.5)
**Hematological malignancies (** ***n*** **, %)**		
no	413 (90.8)	92 (86.0)
yes	42 (9.2)	15 (14.0)
**Congenital heart disease (** ***n*** **, %)**		
no	440 (96.7)	107 (100.0)
yes	15 (3.3)	0 (0.0)
**Hepatitis B cirrhosis (** ***n*** **, %)**		
no	437 (96.0)	105 (98.1)
yes	18 (4.0)	2 (1.9)
**Nephrotic syndrome (** ***n*** **, %)**		
no	448 (98.5)	104 (97.2)
yes	7 (1.5)	3 (2.8)
**Connective tissue disease (** ***n*** **, %)**		
no	446 (98.0)	105 (98.1)
yes	9 (2.0)	2 (1.9)
**Shock (** ***n*** **, %)**		
no	439 (96.5)	23 (21.5)
yes	16 (3.5)	84 (78.5)
**Respiratory failure (** ***n*** **, %)**		
no	424 (93.2)	13 (12.1)
yes	31 (6.8)	94 (87.9)
Total hospitalization time	20 (11.0, 33.0)	7.0 (3.0, 75.0)
Alkaline phosphatase (U/L)	89 (64.0, 137.3)	95.0 (63.5, 148.4)
Alanine aminotransferase (U/L)	22 (13.0, 40.0)	31.0 (16.0, 56.0)
Aspartate aminotransferase (U/L)	23 (7.0, 42.0)	48.0 (24.4, 111.0)
Total protein (g/L)	63.6 (57.9, 69.4)	57.6 (50.5, 68.8)
Albumin (g/L)	34.6 (29.5, 39.4)	27.8 (24.5, 36.9)
Total bilirubin (umol/L)	10.7 (6.8, 17.0)	14.8 (8.0, 30.6)
Direct bilirubin (umol/L)	5.4 (3.1, 8.6)	8.3 (4.0, 15.8)
Indirect bilirubin (umol/L)	4.5 (2.9, 7.4)	4.7 (2.8, 9.4)
Creatinine (umol/L)	61 (49.0, 77.0)	73.0 (55.8, 114.0)
White blood cell	8.3 (5.4, 12.6)	7.6 (2.4, 13.0)
Granulocyte (umol/L)	6.4 (3.6, 10.2)	5.2 (1.4, 10.6)
Ratio of Granulocyte	77.6 (66.1, 87.8)	83.3 (65.3, 93.0)
Fibrinogen (g/L)	4.1 (3.4, 4.9)	3.5 (2.5, 4.4)
D-dimer (mg/L)	0.6 (0.3, 1.3)	2.3 (0.7, 5.5)
Platelet	178.0 (111.0, 253.0)	90.0 (36.0, 176.0)
C-reactive protein	68.9 (25.9, 120.7)	116.7 (50.6, 200.0)

*$\bar{x}$, mean; SD, standard deviation*.

### Different Types of Antibiotics Used by Patients With BSI

Of the 562 patients with BSI, 373 patients were treated with a single antibiotic, and 189 patients were treated with a combination of antibiotics. The results of the chi-square test analysis showed that the mortality rate of patients using a combination of antibiotics was significantly higher than that of patients using a single antibiotic (25.40 and 15.82%, respectively; Table 2). This study did not support the combined treatment of streptococcus bloodstream infection. The seven most commonly used single antibiotics included the following: β-lactam addase inhibitor, carbapenems, cephalosporins, quinolones, glycopeptides, oxazolidinones, fourth generation cephalosporin, penicillins, and tetracyclines (Table 3). Analysis of the mortality of the three most commonly used single antibiotics revealed that the mortality of the β-Lactam addase inhibitor (10.29%) was significantly lower than that of carbapenems (25.49%; *P* = 0.002; Table 4). Among the combined antibiotics used, the top five most commonly used were carbapenem + glycopeptides, β- lactam plus enzyme inhibitor + quinolones, carbapenem + oxazolidinones, cephalosporins + quinolones and β-lactam plus enzyme inhibitor + glycopeptides (Table 4).

**Table 2:** Comparison of mortality of patients with blood stream infection treated with single and combined antibiotics.

	**Survival (*n*, %)**	**Death (*n*, %)**
Single antibiotic	314 (84.18)	59 (15.82)
Combined antibiotics	141 (74.60)	48 (25.40)
χ^2^	7.467	
*P*	0.006	

**Table 3:** Mortality of patients with bloodstream infection treated with different antibiotics.

**Single antibiotic (*n* = 373)**	**Survival (*n*, %)**	**Death (*n*, %)**
β Lactam addase inhibitor	122 (89.71)	14 (10.29)
Carbapenems	76 (74.51)	26 (25.49)
Cephalosporins	71 (85.54)	12 (14.46)
Quinolones	22 (100.00)	0 (0.00)
Glycopeptides	9 (90.00)	1 (10.00)
Oxazolidinones	6 (66.67)	3 (33.33)
Fourth generation cephalosporin	2 (40.00)	3 (60.00)
Penicillins	4 (100.00)	0 (0.00)
Tetracyclines	2 (100.00)	0 (0.00)
**Combined antibiotics (*****n*** **=** **189)**		
Carbapenem + glycopeptides	32 (62.75)	19 (37.25)
β Lactam plus enzyme inhibitor + quinolones	38 (88.37)	5 (11.63)
Carbapenem + oxazolidinones	9 (60.00)	6 (40.00)
Cephalosporins + quinolones	10 (76.92)	3 (23.08)
β Lactam plus enzyme inhibitor + glycopeptides	11 (84.62)	2 (15.38)
Cephalosporins + glycopeptides	11 (91.67)	1 (8.33)
β Lactam plus enzyme inhibitors + oxazolidinones	8 (80.00)	2 (20.00)
Carbapenem + quinolones	8 (88.89)	1 (11.11)
Quinolones + glycopeptides	5 (83.33)	1 (16.67)
Tetracycline + carbapenems	0 (0.00)	5 (100.00)
Penicillin + quinolones	4 (100.00)	0 (0.00)
Fourth generation cephalosporins + oxazolidinones	1 (50.00)	1 (50.00)
Quinolones + oxazolidinones	1 (50.00)	1 (50.00)
Cephalosporins + oxazolidinones	1 (100.00)	0 (0.00)
Cephalosporins + tetracyclines	0 (0.00)	1 (100.00)
Tetracycline + glycopeptides	1 (100.00)	0 (0.00)
Fourth generation cephalosporins + glycopeptides	1 (100.00)	0 (0.00)

**Table 4:** Comparison of mortality of patients with bloodstream infection treated with a common single antibiotic.

**Single antibiotic**	**χ^2^**	***P***
Cephalosporins vs. β Lactam addase inhibitor	0.854	0.355
Cephalosporins vs. Carbapenems	3.413	0.065
β Lactam addase inhibitor vs. Carbapenems	9.626	0.002^*^

* *P < 0.05*.

### Prognostic Factors Associated With Streptococcus Bloodstream Infection

The Kolmogorov–Smirnova analysis was performed to test the normality of continuous data. The results showed that the data did not conform to the normal distribution (*P* < 0.05). Therefore, we applied univariate analysis to explore the potential prognostic factors of patients with BSI (Table 5). Compared with the survival group, patients in the death group were older (*Z* = 3.40, *P* = 0.001); with lower levels of total protein (*Z* = 3.95, *P* < 0.001), albumin (*Z* = 5.21, *P* < 0.001), fibrinogen (*Z* = 4.25, *P* < 0.001), and platelet (*Z* = 6.29, *P* < 0.001); with higher levels of alanine aminotransferase (*Z* = 3.40, *P* = 0.001), aspartate aminotransferase (*Z* = 6.71, *P* < 0.001), total bilirubin (*Z* = 3.71, *P* < 0.001), direct bilirubin (*Z* = 4.16, *P* < 0.001), creatinine (*Z* = 4.13, *P* < 0.001), ratio of granulocytes (*Z* = 2.76, *P* = 0.006), D-dimer (*Z* = 7.64, *P* < 0.001), and C-reactive protein (*Z* = 4.46, *P* < 0.001). Meanwhile, the incidence of diabetes (χ^2^ = 10.46, *P* = 0.001), non-hematological malignancies (χ^2^ = 8.92, *P* = 0.003), respiratory failure (χ^2^ = 328.94, *P* < 0.001), and shock (χ^2^ = 333.02, *P* < 0.001) was higher in the death group. Moreover, the total hospitalization time (*Z* = 6.94, *P* < 0.001) was shorter, and the proportion of men (χ^2^ = 4.65, *P* = 0.031) was higher in the death group. Furthermore, 19 factors with statistical significance in univariate analysis were used as independent variables, and the 60-day outcome of patients with BSI were used as dependent variables for the multivariate logistic regression analysis (Table 6). The results indicated that shock (OR = 16.61, 95%CI: 7.00–39.41) and respiratory failure (OR = 47.53, 95%CI: 19.93–133.64) were independent risk factors of 60-day death, and higher levels of albumin (OR = 0.94, 95%CI: 0.89–0.99) and fibrinogen (OR = 0.61, 95%CI: 0.46–0.82) were independent protective factors for patients with BSI.

**Table 5:** Univariate analysis of patients with streptococcal bloodstream infection.

**Variables**	**Z/χ^2^**	***P***
Sex	4.65	0.031
Year	3.40	0.001
Hypertension	2.41	0.120
Diabetes	10.46	0.001
Coronary heart disease	0.01	0.911
Malignant tumor (non-blood system)	8.92	0.003
Hematological malignancies	2.18	0.140
Congenital heart disease	2.47	0.116
Hepatitis B cirrhosis	0.58	0.448
Nephrotic syndrome	0.24	0.628
Connective tissue disease	0.01	0.942
Total hospitalization time	6.94	0.000
Alkaline phosphatase (U/L)	0.45	0.654
Alanine aminotransferase (U/L)	3.40	0.001
Aspartate aminotransferase (U/L)	6.71	0.000
Total protein (g/L)	3.95	0.000
Albumin (g/L)	5.21	0.000
Total bilirubin (umol/L)	3.71	0.000
Direct bilirubin (umol/L)	4.16	0.000
Indirect bilirubin (umol/L)	0.90	0.369
Creatinine (umol/L)	4.13	0.000
White blood cell	1.26	0.207
Granulocyte	1.31	0.190
Ratio of Granulocyte	2.76	0.006
Fibrinogen	4.25	0.000
D-dimer	7.64	0.000
Platelet	6.29	0.000
C-reactive protein	4.46	0.000
Shock	333.02	0.000
Respiratory failure	328.94	0.000

**Table 6:** Multivariate analysis of patients with streptococcal bloodstream infection.

**Variable**	**β**	**SE**	**OR**	**95%CI**	***P***
Albumin	−0.058	0.026	0.94	0.89–0.99	0.027
Fibrinogen	−0.489	0.146	0.61	0.46–0.82	0.001
Shock	2.810	0.441	16.61	7.00–39.41	0.000
Respiratory failure	3.861	0.455	47.53	19.93–133.64	0.000

### Survival Analysis of Patients With BSI in Prognostic Factors

**Figure 2:**
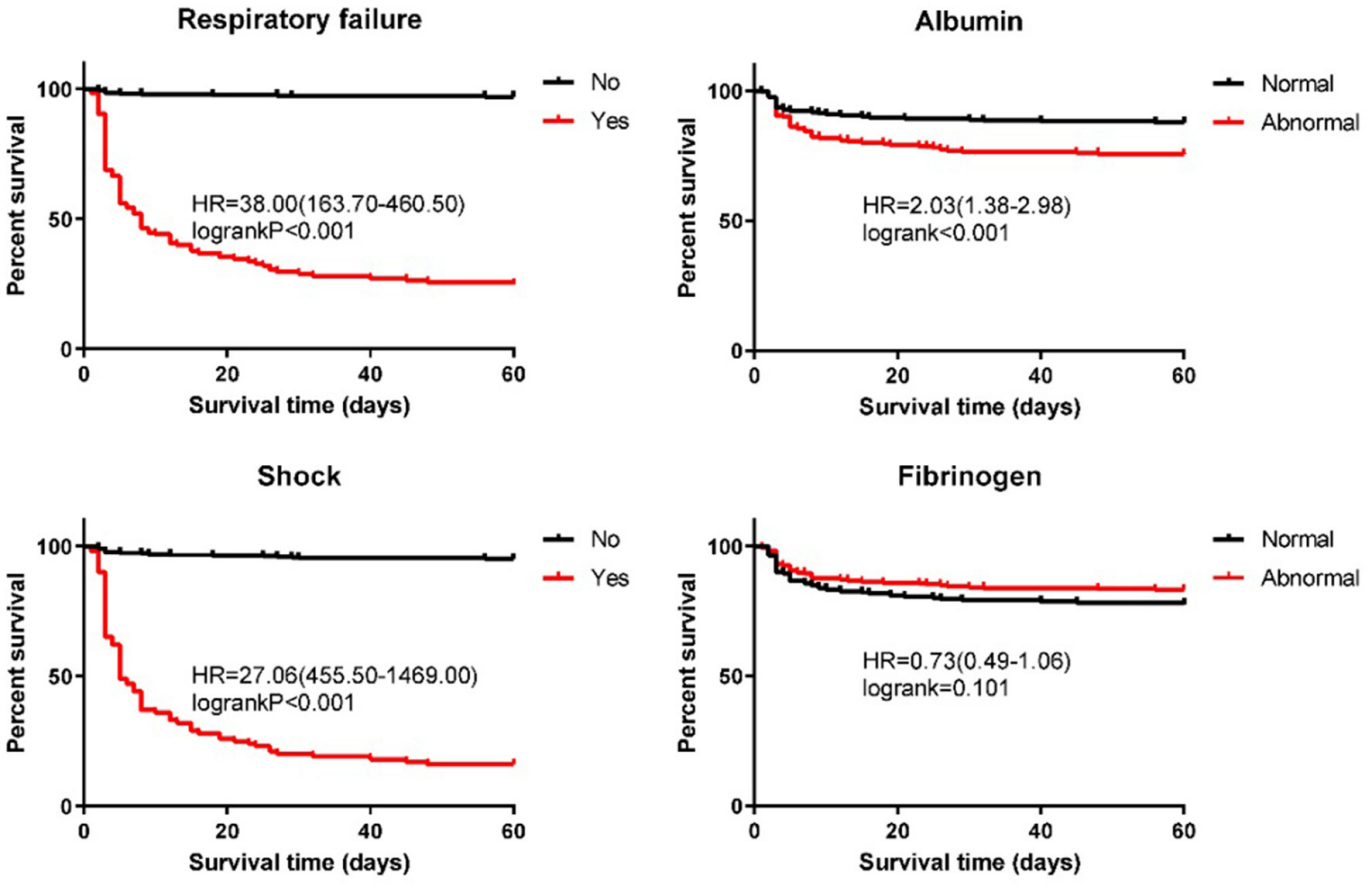
Kaplan–Meier estimates of the overall survival for patients with streptococcus bloodstream infection.

Further, to test the four independent prognostic factors (shock, respiratory failure, albumin, and fibrinogen), we used the log-rank test to make a survival analysis on patients with BSI. The median survival time of 562 patients was 60 days, and the 60-day cumulative survival rate was 81%. We defined the albumin range of 35–55 g/L and the fibrinogen range of 2–4 g/L as normal. The survival analysis of patients with BSI in terms of prognostic factors is presented in Figure 2. At the time of this analysis, the cumulative survival rates of patients without and with shock were 95% (median OS, 60 days) and 16% (median OS, 5 days), respectively (χ^2^ = 478.04, *P* < 0.001). Patients without respiratory failure had a better prognosis than patients with respiratory failure (97 vs. 25%), and the difference was statistically significant (χ^2^ = 431.79, *P* < 0.001). For patients without and with respiratory failure, the median survival times were 60 and 8 days, respectively. In terms of albumin level, the cumulative survival rate of patients with a normal level was significantly higher than that of patients with an abnormal level (88 vs. 76%; median OS: 60 vs. 60 days; χ^2^ = 13.00, *P* < 0.001). As can be seen from the figure, the normal level of fibrinogen has a negative effect on prognosis, but there was no statistical significance in the difference (χ^2^ = 2.69, *P* = 0.101).

### Predictive Value of Different Prognostic Factors for Patients With BSI

**Figure 3:**
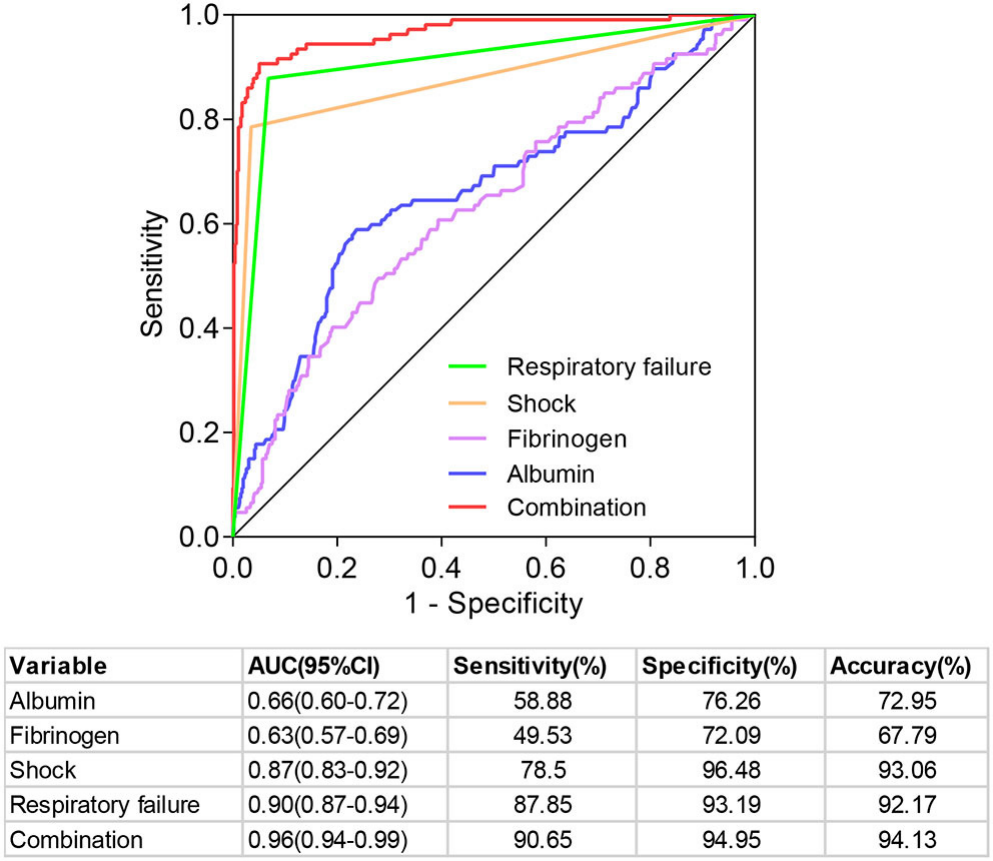
Predictive value of different prognostic indicators in predicting the prognosis of patients with bloodstream infection.

Since shock, respiratory failure, albumin, and fibrinogen were independent prognostic factors, we further analyzed the predictive value of four indicators and their combination for the prognosis of patients with BSI (Figure 3). The ranges of sensitivity and specificity of the four factors were 49.53–87.85% and 72.09–96.48%, respectively. The area under the ROC (AUC) is a comprehensive evaluation index to measure the accuracy of the binary classification model. The AUCs of the four factors ranged from 0.66 (95%CI: 0.60–0.72) to 0.90 (95%CI: 0.87–0.94). Moreover, the results demonstrated that the combination of the four prognostic indicators had a high predictive value for the 60-day outcome of patients with BSI, with an AUC of 0.96 (95%CI: 0.94–0.99), sensitivity of 90.65%, specificity of 94.95%, positive likelihood ratio of 17.95, negative likelihood ratio of 0.10, and accuracy of 94.13%.

## Discussion

Among the 562 BSI patients included in this study, more patients used a single antibiotic (66.37%) than the combined antibiotics (33.63%), and the most commonly used single antibiotics and combined antibiotics were β-lactam addase inhibitor and carbapenem + glycopeptides, respectively. It is worth noting that the mortality was higher in patients treated with a combination of antibiotics (25.40%) than patients treated with a single antibiotic (15.82%), perhaps because patients treated with combined antibiotics had more serious cases. Additionally, the physician in charge believed that the patients were co-infected with other bacterial infections, although there were no other positive etiological results.

In the present study, significant factors associated with the 60-day mortality of patients with BSI in the univariate analysis included older age, male, shorter hospitalization stay, diabetes, non-hematological malignancies, shock, respiratory failure; lower total protein, albumin, fibrinogen, and platelet levels; and higher alanine aminotransferase, aspartate aminotransferase, total bilirubin, direct bilirubin, creatinine, ratio of granulocytes, D-dimer, and C-reactive protein levels. After adjusting for possible confounding factors, a prognostic model consisting of shock, respiratory failure, albumin, and fibrinogen was established for predicting the outcome of patients with BSI. Most diseases were age-related; in our current research, patients who died had a higher average age (51.4 ± 25.0 vs. 44.4 ± 22.1 years; *P* = 0.001), which is consistent with research by Kevin et al. (10). Cancer is the main cause of deterioration of clinical outcomes in patients with invasive streptococcosis (11, 12). Our data showed more patients with malignancy in the death group (21.5 vs. 10.8%; *P* = 0.003), which also supported previous reports. Notably, our results suggested that the proportion of patients with diabetes in the death group was higher than that in the survival group (21.5 vs. 10.1%; *P* = 0.001). In a multinational population-based assessment of group B streptococcus BSI, diabetes was the most common comorbidity (13).

Among these four factors that were determined to be crucial survival factors for patients with BSI, higher albumin and fibrinogen levels might be protective indicators for prognosis. A recent report on inflammatory biomarkers in patients with BSI demonstrated that lower albumin levels in neutropenic BSI indicate a poorer infection prognosis (14). Albumin, accounting for 55% of plasma protein, is completely synthesized by the liver and has a long half-life of ~20 days (15). Albumin has several important functions, including material binding, material transportation, enzyme activity, and antioxidation (16). It can regulate the main signal of inflammatory cells according to the redox state, which reduces body inflammation, protects organ function, and reduces acute kidney injury (17). Studies have confirmed that human serum albumin can achieve an ideal effect in the treatment of sepsis (18). Fibrinogen is a pleiotropic protein that plays an important role in blood coagulation, inflammation, and tissue repair and can alter many aspects of inflammatory cell function by involving white blood cells through various cell receptors and mechanisms (19, 20). Studies have shown that *S. aureus* can produce the virulence factor Efb in forming a “fibrinogen barrier” to inhibit platelet activation and phagocytosis of innate immune cells (21, 22). Mo et al. reported that pulmonary infection caused coagulation abnormality in the body, and various coagulation factors were gradually consumed in the process of thrombosis, so the levels of coagulation factors and fibrinogen decreased (23).

Sepsis and bacteremia are called BSIs. Severe sepsis can cause shock, disseminated intravascular coagulation, and multiple organ failure. In this study, shock and respiratory failure were risk indicators for prognosis in patients with BSI. A study on *Klebsiella pneumoniae* BSI and predictors of mortality pointed out that septic shock (OR:6.42, 95% CI: 1.34–30.69) was an independent risk factor for 28-day mortality of *K. pneumoniae* BSI (24). A report on risk factors and outcomes in *K. pneumoniae* BSIs of 370 patients revealed that respiratory failure (OR:5.27, 95% CI: 1.40–19.87) was independently associated with higher mortality risk (25).

This study used the logistic regression to construct the prediction model of patients with BSI. It is a supervised statistical learning method and has the great advantage that the prediction results are output in the form of probability. In addition, the interpretability of the model is very simple. At present, there are many new modeling methods, such as ensemble modeling methods that can address non-linearity automatically without pre-specification (26). This study is the first to establish a prediction model related to the prognosis of patients with streptococcus BSI, which achieved high accuracy. This study also has certain limitations. Due to the existence of missing data, the blood lipid data of the study subjects were not included in the analysis. Additionally, this study was a single-center retrospective study. Therefore, sampling error and confounding factors may affect the outcome, which needs to be confirmed by further multicenter randomized controlled clinical studies.

In conclusion, this study demonstrated that shock, respiratory failure, albumin, and fibrinogen are independent prognostic factors for 60-day mortality, and the appropriate treatment measures should be taken in light of these prognostic factors to improve the survival rate of patients with BSI.

## Data Availability Statement

The raw data supporting the conclusions of this article will be made available by the authors, without undue reservation.

## Ethics Statement

The studies involving human participants were reviewed and approved by the Medical Ethics Committee of the First Affiliated Hospital of Zhengzhou University. Written informed consent from the participants or their legal guardian/next of kin was not required to participate in this study in accordance with the national legislation and the institutional requirements.

## Author Contributions

XD and TS contributed to conception and design of the study, wrote the first draft of the manuscript and wrote sections of the manuscript. RZ, XZ, and XD organized the database. XD performed the statistical analysis. All authors contributed to manuscript revision, read, and approved the submitted version.

## Funding

This study was supported by the United Fund of National Natural Science Foundation of China (Grant No. U2004110), the National Natural Science Foundation of China (Grant No. 82172129).

## Conflict of Interest

The authors declare that the research was conducted in the absence of any commercial or financial relationships that could be construed as a potential conflict of interest.

## Publisher's Note

All claims expressed in this article are solely those of the authors and do not necessarily represent those of their affiliated organizations, or those of the publisher, the editors and the reviewers. Any product that may be evaluated in this article, or claim that may be made by its manufacturer, is not guaranteed or endorsed by the publisher.
